# Complete Genome Sequence of *Caulobacter* sp. (Strain NIBR1757) Isolated from Lake Chungju in South Korea

**DOI:** 10.1128/mra.00086-23

**Published:** 2023-06-12

**Authors:** Se-Young Kim, Jin Young Park, Jaewoong Yu, Jin Nam Kim, Ahhyeon Choi

**Affiliations:** a College of Agriculture and Life Sciences, Seoul National University, Seoul, Republic of Korea; b Biological Resources Research Department, National Institute of Biological Resources, Incheon, Republic of Korea; c eGnome, Inc., Seoul, Republic of Korea; University of Southern California

## Abstract

Here, we report the complete genome sequence of strain NIBR1757, isolated from the water of Lake Chungju in South Korea. The assembled genome consists of 4,185 coding sequences (CDSs), 6 rRNAs, and 51 tRNAs. Comparative 16S rRNA gene sequence studies and GTDB-Tk analysis show that this strain belongs to the genus *Caulobacter*.

## ANNOUNCEMENT

Lake Chungju is one of the largest artificial lakes in South Korea used as a water source (1, 2). Microorganisms in freshwater serve as indicators of water quality and can also function as indicators of global climate change, due to their significant impact on carbon cycling and the biological communities of freshwater environments (3–6). We investigate aquatic microorganisms and intend to use them as a basis for studying the aquatic ecosystems of Lake Chungju.

Strain NIBR1757 was isolated from surface water (depth, 0 to 30 cm) of Lake Chungju in South Korea (37°00′43.2″N, 128°10′10.0″E). The freshwater sample was serially diluted, spread onto Reasoner’s 2A (R2A) agar, and incubated at 25°C for 3 days. To obtain a pure colony, the yellow colonies were streaked onto R2A agar. Strain NIBR1757 was cultivated in 100 mL of R2A broth with agitation (100 rpm) at 25°C for 3 days. Genomic DNA was extracted using the RBC DNA extraction kit (Taiwan), sheared using a g-TUBE device (Covaris, USA), and purified using 0.45× AMPure XP magnetic beads (Beckman Coulter, Inc.). A library was prepared using the PacBio SMRTbell library preparation kit ver. 1.0 (Pacific Biosciences, Menlo Park, USA). DNA damage repair, end repair, and ligation were performed following the protocol provided in the kit. The library was cleaned up using the SMRTbell enzyme cleanup kit (Pacific Biosciences), and size selection was performed using the Blue Pippin system (Sage Science, Beverly, USA) with a cutoff of 15 kb.

Sequencing was performed using the PacBio RS II platform, and a total of 2,372,912 reads were obtained, with an *N*_50_ value of 9,446 bp. Preassembly read quality control was performed using NanoPlot ver. 1.40.0 (7), and reads shorter than 1,000 bp were removed using SeqKit ver. 2.3.0 (8). *De novo* assembly of the reads was conducted using Flye ver. 2.9 (9) with the “–meta” option. Default parameters were used for all software unless otherwise specified. The quality of the assembled contig was evaluated using QUAST ver. 5.2 (10) and BUSCO ver. 5.2.2 (11). The completeness was 99.2%, evaluated using BUSCO with bacteria_odb10 as the lineage data set. For circularization, Circlator ver. 1.5.5 (12) was used to trim the overlapping end and rotate the genome to fix the start position at the *dnaA* gene.

The complete genome of NIBR1757 consists of a circular chromosome of 4,470,728 bp with 3,619× coverage. The GC content accounted for 68.3% of the whole genome. The complete genome sequence of NIBR1757 was annotated using Prokka ver. 1.14.6 (13). It has 4,185 coding sequences, of which 6 coding DNA sequences (CDSs) were suggested to encode rRNAs and 51 CDSs were suggested to encode tRNAs. Using the BLASTn program, the 16S rRNA gene of NIBR1757 was confirmed to be 99.79% identical to that of Caulobacter fusiformis ATCC 15257 (GenBank accession number NR_025320.1). Additionally, Genome Taxonomy Database Toolkit (GTDB-Tk) (14) analysis and average nucleotide identity (ANI) calculation using Pyani ver. 0.2.12 (15) were performed. Using GTDB-Tk ver. 1.5.0, NIBR1727 was assigned to the genus *Caulobacter*, but it could not be identified at the species level. ANI analysis confirmed that NIBR1757 had the highest ANI value with *Caulobacter* sp. strain SLTY (GCF_009905535.1), at 82%.

### Data availability.

The complete genome sequence of *Caulobacter* sp. strain NIBR1757 has been deposited at GenBank under the accession number CP115463. The raw data have been deposited in the SRA under the accession number SRR22875672.
